# Microglia Polarization: A Novel Target of Exosome for Stroke Treatment

**DOI:** 10.3389/fcell.2022.842320

**Published:** 2022-03-09

**Authors:** Teng Wan, Yunling Huang, Xiaoyu Gao, Wanpeng Wu, Weiming Guo

**Affiliations:** ^1^ Hengyang Medical College, University of South China, Hengyang, China; ^2^ Sports Medicine Department, Huazhong University of Science and Technology Union Shenzhen Hospital, The 6th Affiliated Hospital of Shenzhen University Health Science Center, Shenzhen, China; ^3^ Shenzhen Futian District Maternity & Child Healthcare Hospital, Shenzhen, China

**Keywords:** exosomes, microglia polarization, stroke treatment, microRNA, neuroinflammation, neuroprotection

## Abstract

The vast majority of cells in the human body are capable of secreting exosomes. Exosomes have become an important vehicle for signaling between cells. Exosomes secreted by different cells have some of the structural and functional properties of that cell and thus have different regulatory functions. A large number of recent experimental studies have shown that exosomes from different sources have different regulatory effects on stroke, and the mechanisms still need to be elucidated. Microglia are core members of central intrinsic immune regulatory cells, which play an important regulatory role in the pathogenesis and progression of stroke. M1 microglia cause neuroinflammation and induce neurotoxic effects, while M2 microglia inhibit neuroinflammation and promote neurogenesis, thus exerting a series of neuroprotective effects. It was found that there is a close link between exosomes and microglia polarization, and that exosome inclusions such as microRNAs play a regulatory role in the M1/M2 polarization of microglia. This research reviews the role of exosomes in the regulation of microglia polarization and reveals their potential value in stroke treatment.

## Introduction

According to a report published by the World Health Organization in 2020, stroke is the second leading cause of death worldwide. The lethal and disabling nature of stroke greatly increases the burden on society and individual families. The main treatments are intravenous thrombolysis and mechanical thrombectomy. Both of therapeutic strategies are limited by the recommended treatment time window and the effect is still not ideal (Thiebaut et al., 2018; Ozaki et al., 2019). In order to achieve better treatment, research on new therapeutic targets is necessary.

Ischemic stroke is the main type of stroke pathogenesis, mainly due to impaired blood supply to the brain, which occurs followed by a series of ischemia-reperfusion injuries, such as inflammation and oxidative stress, while persistent neuroinflammation damages neurons and the blood-brain barrier, which in turn leads to disease progression (Zhuang et al., 2017; Xu et al., 2018; Azedi et al., 2019). In the pathological process of stroke, injury are mainly induced by ischemia and/or ischemia-reperfusion, and microglia play an important role in this process. When microglia are activated, they transform into two main M1 and M2 phenotypes. M1 is the pro-inflammatory type, which secretes substances such as interleukin-1*β* (IL-1*β*), interleukin-6 (IL-6), and tumor necrosis factor (TNF) that promote inflammation and neurotoxic substances that aggravate brain damage (Shu et al., 2016; Zhu et al., 2019). M2 phenotype is anti-inflammatory, which secretes anti-inflammatory cytokines such as interleukin-4 (IL-4), interleukin-10 (IL-10), transforming growth factor-*β* (TGF-*β*) and some neurotrophic factors, which facilitate brain function recovery and improve the prognosis of stroke (Hu et al., 2012; Liu et al., 2016; Shu et al., 2016).

Exosomes are involved in cell-to-cell regulation through their various inclusions (Braccioli et al., 2014). It not only regulates normal physiological processes, but also assumes an important role in the development of some diseases such as cancer (Pan and Johnstone, 1983). According to recent studies, it was found that exosome transport of miR-124-3p, a substance that promotes M2 microglial polarization, reduced brain damage and improved the outcome of stroke (Huang et al., 2018). This study will summarize the mechanisms of communications between microglia and stroke and elucidate roles of different sources of exosomes in regulating stroke by targeting microglia polarization, thus providing new therapeutic strategies for stroke.

## Exosome

Almost all prokaryotes and eukaryotes can release extracellular vesicles (EVs) for communication with other cells (Kalluri and LeBleu, 2020). The classification of EVs is constantly improving. Currently, the EVs are mainly divided into two categories: ectosome and exosome (Cocucci and Meldolesi, 2015; Théry et al., 2018). Exosome is a special type of secretory vesicle with small volume in EVs, which is wrapped with a phospholipid bilayer outside. And its diameter is about 50 nm to 1 um (Kalluri and LeBleu, 2020). Exosomes contain proteins, lipids, deoxyribonucleic acid, messenger RNA (mRNA), microRNA and so on (Braccioli et al., 2014). Exosomes emerge from the plasma membrane and endosomal membrane, diffuse into the intercellular fluid, fuse with the recipient cells, release their contents, and exert regulatory effects (Mittelbrunn and Sánchez-Madrid, 2012). they can be produced from a variety of cells such as reticulocytes, antigen-presenting cells, tumor cells, skeletal cells, etc., and the exosomes secreted by different cells have different biological effects and different intensity of effects (Harding et al., 1983; Anderson et al., 2005; Braccioli et al., 2014) (Fordjour et al., 2019). In terms of morphological structure, the exosomes have phosphatidylethanolamine (PE) and phosphatidylserine (PS) on the bilayer membrane, which is a distinctive feature of the exosome plasma membrane and also makes the exosomes have a high membrane curvature, which is conducive to maintaining the stability of the exosome membrane (Booth et al., 2006).

Exosome-mediated cell-to-cell interaction involves many physiological processes such as reproduction and development of mammals, immune response, metabolism, and many pathological changes such as neurodegenerative diseases, cardiovascular diseases and tumorigenesis (Kalluri and LeBleu, 2020). Exosomes play an important role in the physiological activities of cells, e.g., during fertilization, Juno receptors on the ovum are shed as exosomes, thus preventing other sperm from fertilizing (Bianchi et al., 2014). Exosomes, which originate from the embryonic trophectoderm, can transmit their characteristic high resistance to viral infection to other cells (Delorme-Axford et al., 2013). Exosomes, with their PS-rich outer membrane, play an important role in the osteogenesis process, and osteoblasts secrete exosomes to initiate the process of mineralization *in vivo* (Anderson et al., 2005). They also have an important regulatory role in the pathological processes of cells. In tumor cells, the exosome secretion pathway is held hostage by tumor cells to accomplish a variety of pathological activities, and exosomes contain substances that stimulate tumor proliferation (Skog et al., 2008). The exosomes contain substances that stimulate tumor proliferation. Metastatic melanoma cells suppress cellular immunity by releasing exosomes with Programmed Cell Death-Ligand 1 (PD-L1) on their surface and upregulating the intracellular PD-L1 concentration in target cells (Chen et al., 2018). HIV virus can also fuse with normal cells in the form of exosomes, thus infecting normal cells (Melikyan, 2014) (Pegtel et al., 2010). In summary, exosomes are used as a means to protect the normal cells. In conclusion, exosomes play an important role in the mutual regulation of cells as messengers of intercellular signaling.

Exosomes have an important role in the development of many diseases, especially neurological diseases. mRNA mutants and miRNAs characteristic of glioma can be detected in serum exosomes of glioblastoma patients, which may become a new target for the diagnosis and treatment of this disease (Skog et al., 2008). Prions are essentially a misfolded form of protein, and their spread *in vivo* is mainly in the form of exosomes (Fevrier et al., 2004). A large number of recent studies have shown the applicability of exosomes for the treatment of stroke. One reason is that exosomes can easily cross the blood-brain barrier and have the potential to be used as a vehicle for brain-targeted modulation or drug administration (Rufino-Ramos et al., 2017; Khan et al., 2021). Second, exosomes were found to transport miRNAs such as miR-124-3p, which can promote microglia polarization toward the anti-inflammatory M2 phenotype, thereby inhibiting neuroinflammation (Huang et al., 2018). These suggest that exosomes may become important therapeutic targets in the stroke.

In view of the heterogeneity of exosomes in source, content, function and size, exosomes have a good prospect in the diagnosis and treatment of different clinical diseases. The research shows that exosomes may become diagnostic markers of many diseases, including cardiovascular diseases (Zhang et al., 2017a; Jansen and Li, 2017), cancer (Fitts et al., 2019), liver-related diseases (Masyuk et al., 2013), central nervous system diseases and so on (Kanninen et al., 2016). In the mouse model, compared with the use of liposomes, exosomes are able to enter cells more effectively with less immune clearance *in vivo* (Ferguson and Nguyen, 2016; Barile and Vassalli, 2017; Liao et al., 2019). Exosomes play an important role in cardiovascular function regulation and cardiovascular and cancer therapy (Li et al., 2021a; Lim, 2021; Nasser et al., 2021; Robson, 2021). With the development of technology, artificial exosomes have been used to deliver various nanomedicines (Li et al., 2021b). Therefore, the clinical transformation of exosomes in the diagnosis and treatment of different diseases deserves further research.

## Exosome and Microglia

### Overview of Microglia

Microglia are resident cells of the Central nervous system (CNS) and belong to the monocyte macrophage system, but microglia, unlike other individuals in the monocyte macrophage system, develop initially from c-KitloCD41lo progenitor cells produced in the yolk sac (YS) around embryonic day 7.25 (E7.25), well before the appearance of other glial cells (Ginhoux et al., 2010). Microglia exhibit different states and have different functions during developmental and adult stages. During development, microglia are “phagocytic” and mobile amoeba-like, reflecting the phagocytic role of microglia in removing dead cells and remodeling neural tissue (Frost and Schafer, 2016; Matcovitch-Natan et al., 2016; Hagemeyer et al., 2017). Microglia have multiple effects on synapses, removing non-functional synapses and remodeling synaptic circuits, as demonstrated by Paolicell et al., 2011, in which C-X3-C motif chemokine receptor 1 (CX3CR) 1-deficient animals exhibit a defective number of microglia and transient defects in synaptic connections during development (Paolicelli et al., 2011; Wake et al., 2013). Microglia at the adult stage appear to be quiescent, but in fact they are constantly scanning and monitoring their surroundings with their characteristic branches and interacting with neighboring cells. Their ability to clear cells is also very strong and does not even require activation (Hambardzumyan et al., 2016).

In the case of stroke, the activated microglia will promote neuroinflammation and thus further brain tissue damage (Jiang et al., 2020). Conversely, induction of M2 microglia polarization facilitates stroke recovery. Multiple pathways can induce different microglia polarization. Among transcription factors, Nuclear factor kappa B (NF-κB), signal transducer and activator of transcription (STAT) family members, and thrombospondin A2R receptor can promote microglial cell conversion to M1 type. Yang et al. used safranin which reduces the expression of M1 markers, inhibits microglial cell conversion to M1 type by reducing IκB*α* phosphorylation and NF-κB/p65 nuclear translocation (Yang et al., 2019a). Elena Butturini et al. showed that signal transducer and activator of transcription 1 (STAT1) gene silencing can counteract the hypoxic-M1 microglia phenotype, thus suggesting that STAT1 can promote microglia polarization to the M1 phenotype (Butturini et al., 2019).

In ischemia/reperfusion mice, increased Thromboxane A2 receptor (TXA2R) expression was detected in microglia/macrophages by double staining with immunofluorescence, and the TXA2R antagonist SQ29548 inhibited M1 microglia activation and subsequent inflammatory response (Yan et al., 2016). The transcription factors nuclear factor erythroid 2-related factor 2 (Nrf2) and translocation of proliferator-activated receptor gamma (PPAR*γ*) promote microglial cell transition to the M2 phenotype (Jiang et al., 2020). It has been shown that l-F001, a novel multifunctional rho-related protein kinase inhibitor, can increase the expression level of the M2 microglia marker CD206 by activating the Nrf2 signaling pathway *in vitro* (Chen et al., 2017). A study by Liu et al. found that 10-o- (n,n-dimethylaminoethyl) -ginkgolide B methanesulfonate, a new derivative of ginkgolide B, could convert polarized BV2 microglia from M1 phenotype to M2 phenotype by promoting the translocation of PPAR*γ* from the nucleus to the cytoplasm (Liu et al., 2018). Multiple ways have been found capable of inducing microglia polarization. At the same time, it has been found that some substances can convert polarized M1 microglia to the M2 phenotype. This suggests that modulating the conversion of microglia to M2 phenotype may be a practicable therapeutic strategy for stroke.

### Exosome-Derived miRNA and Microglia Polarization

Recent studies have shown that different miRNAs transported by exosomes contribute to the polarization of microglia into different phenotypes. miRNAs are endogenous hairpin-loop structured non-coding RNAs, which mainly bind to mRNAs to block their translation and regulate gene expression. Exosomes containing miR-192-5p, miR-183, miR-378, miR-140-3p, miR-222 were found to inhibit the expression of TNF or/and IL-1*β* expression, thereby promoting microglia M2 polarization (Das et al., 2014; Ti et al., 2015; Eirin et al., 2017; Thomi et al., 2019; Bian et al., 2020). NF-κB is an important signaling hub that drives microglia M1 polarization (Taetzsch et al., 2015). It has been reported that a variety of miRNAs contained in exosomes can regulate the expression of microglia Toll-like receptors (TLRs), thus acting on NF-κB to regulate microglia polarization (Hajinejad and Sahab-Negah, 2021). miR-223, miR-101-3p, miR-26a -5p, miR-326, miR-182, miR-17-5p, miR-140-5p, miR-9, miR-let7, and miR-181c play a role in inhibiting M1 microglia polarization by downregulating TLR expression including TLR2 and TLR4 (Kumar et al., 2015; Wang et al., 2015; Li et al., 2016; Li et al., 2017; Qian et al., 2017; Li et al., 2019; Li et al., 2020a; Yang et al., 2020a; Chaurasiya et al., 2020; Huang et al., 2020). In addition, miR-26b-5p inhibited the TLR signaling pathway by regulating the reduction of CH25H protein expression, which in turn inhibited microglia M1 polarization (Li et al., 2020b).

In addition to regulating TLR expression, miRNAs can also directly regulate NF-κB expression levels. Studies have reported that miR-34a reduces the secretion of pro-inflammatory cytokines such as TNF and IL-1*β* by decreasing the expression of NF-κB and Notch-1 proteins (Domenis et al., 2018). miR-21 reduces the expression of TNF by inhibiting NF-κB pathway, increases the production of anti-inflammatory factor IL-10 and promotes microglia polarization to M2 phenotype (Das et al., 2014). miR-146a-5p downregulates the expression of M1-type microglia-related genes by inhibiting Interleukin-1 receptor-associated kinases (IRAK1) and Nuclear factor of activated T cells 5 (NFAT5) and thus downregulating the expression of genes characteristic of M1-polarized microglia (Duan et al., 2020). miRNAs have also been found to regulate both TLR and NF-κB expression. miR-216-3p shifts microglia from M1 to M2 phenotype by inhibiting the TLR/NF-κB signaling pathway (Liu et al., 2020). In addition, exosomes containing miR-21-5p and miR-29b-3p promote microglia polarization from M1 to M2 phenotype and inhibit M1 polarization by suppressing Inducible nitric oxide synthase (iNOS) mRNA expression and reducing the production of pro-inflammatory factors (Tsai et al., 2019; Jiang et al., 2020). It was found that miR-124 promoted microglia polarization to M2 type by inhibiting the transcription factor CCAAT/enhancer binding protein-*α* (C/EBP-*α*) and its downstream target protein Purine Rich Box-1 (PU.1) (Zha et al., 2021).

**FIGURE 1:**
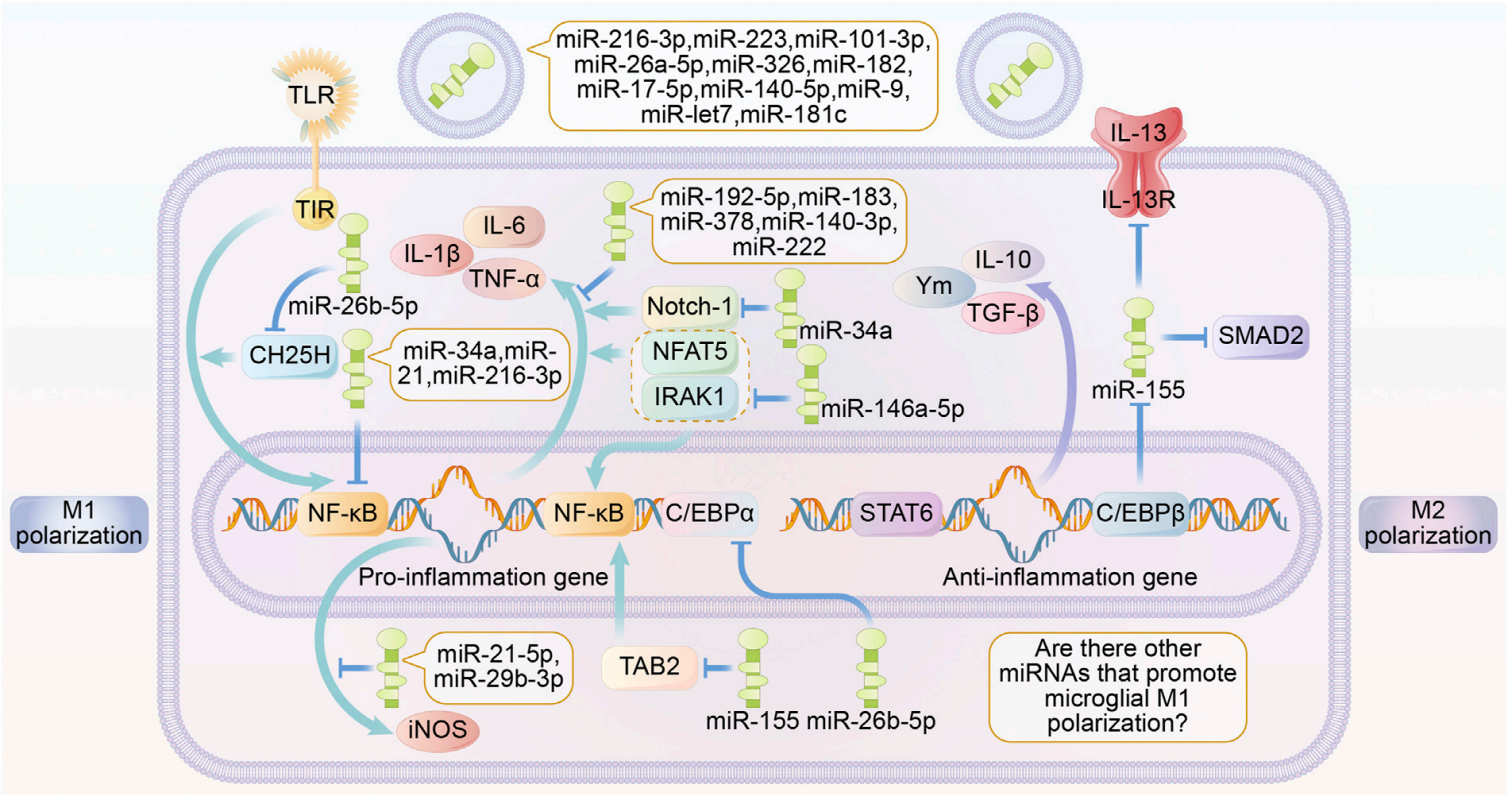
Roles of exosome miRNA in microglia polarization. Different miRNAs secreted by exosomes are able to play a role in regulating microglia polarization through multiple signaling pathways. miR-216-3p, miR-223, miR-101-3p, miR-26a-5p, miR-326, miR-182, miR-17-5p, miR-140-5p, miR-9, miR-let7, miR-181c are able to inhibit TLR expression. miR-192-5p, miR-183, miR-378, miR-140-3p, miR -222 are able to directly inhibit the expression of inflammatory factors. miR-26b-5p inhibits the TLR signaling pathway by suppressing the expression of CH25H. miR-34a, miR-21, miR-216-3p are able to directly inhibit the expression of NF-κB. miR—21-5p, and miR-29b-3p inhibits the expression of the pro-inflammatory factor iNOS. miR-155 inhibits NF-κB activation by suppressing TAB2. miR-26b-5p inhibits C/EBP*α* expression. miR-146a-5p inhibits the expression of IRAK1 and NFAT5, thereby suppressing the expression of inflammation-associated genes and products. miR-34a inhibits the expression of Notch-κB. MiR-34a is able to inhibit Notch-1. The above miRNAs promote microglia M2 polarization and anti-inflammatory factor production. miR-155 promotes microglia M1 polarization and inflammatory factor production by inhibiting the expression of IL-13 receptor, SMAD2 and C/EBP*β*. Abbreviations: TLR, toll like receptor; iNOS, inducible nitric oxide synthase; TGF *β*, transforming growth factor *β*; Ym, chitinase-like proteins; C/EBP*α*, CCAAT/enhancer-binding protein *α*; C/EBP*β*, CCAAT/enhancer-binding protein *β*; TNF-*α*, tumour necrosis factor *α*; STAT6, signal transducer and activator of transcription 6; NF-κB, Nuclear factor kappa B; NFAT5, nuclear factor of activated T cells 5; IRAK1, interleukin-1 receptor-associated kinase1; TAB2, TGF-Beta-Activated Kinase 1-Binding Protein 2; SMAD2, Sma- And Mad-Related Protein 2.

As mentioned above, exosomal miRNAs that act on the TLR/NF-κB pathway and its downstream signaling pathway can inhibit microglia M1 polarization. Uniquely, miR-155 was reported to promote M1 polarization in microglia. The mechanism may be related to the suppression of IL-13R, SMAD2 and CCAAT/enhancer-binding protein *β* (CEBP*β*) expression (Allen et al., 2013; Dickey et al., 2017). However, miR-155 has been reported to promote M2 polarization by inhibiting TGF-Beta-Activated Kinase 1-Binding Protein 2 (TAB2) (Zhang et al., 2018). Most studies have reported the inhibitory roles of miRNAs in M1 polarization, while studies on miRNAs that inhibit M2 polarization-related pathways are still lacking and deserve further exploration (Figure 1).

## Microglia in Stroke

### Microglia and Neuroinflammation

Acute cerebral ischemia is the initiating event of stroke. Inadequate blood supply leads to hypoxia and glucose shortage, disruption of homeostasis, which leads to inflammation, glutamate excitotoxicity, and mitochondrial dysfunction (Mo et al., 2020). Post-ischemia inflammation is mainly caused by necrotic tissue and activated inflammatory cells (Zhang et al., 2020). Microglia are the major intrinsic immune cells of the CNS and dominate the regulation of central inflammation. Damps (damage-associated molecular proteins) include high mobility group box 1tbox1 protein (HMGB-1), extracellular peroxiredoxin (Prx) family proteins and galectin-3 (Gal3), which are closely associated with microglia activation (Amruta et al., 2020). During stroke, hypoxic necrosis of brain cells leads to the production of various DAMPs (Xiong et al., 2016). HMGB-1 stabilizes nucleosome structure and participates in several physiological processes as a signaling factor. In stroke, HMGB-1 acts as a pro-inflammatory cytokine that activates microglia through the receptor of advanced glycation end-product (RAGE) and TLR-MyD88 pathways, producing immunosuppressive and lymphotoxic effects that further aggravate brain injury (Liesz et al., 2015; Jayaraj et al., 2019). Lin et al. found that in a mouse model of cerebral hemorrhage, heme activates TLR4 which in turn activates the MyD88/TRIF signaling pathway, ultimately allowing activation of the NF-κB pathway (Lin et al., 2012). Activated M1 microglia can release pro-inflammatory cytokines, reactive oxygen species (ROS) and Matrix metalloproteinase (MMP) to disrupt the blood-brain barrier and exacerbate neuroinflammation. After necrotic tissue is cleared, M2 microglia secrete the anti-inflammatory cytokine IL-10 and the neurotrophic factor IGF-1 to promote neural repair and reduce brain damage (Iadecola and Anrather, 2011). High expression of CD206, a marker of M2, and high expression of MHCII, a marker of M1, have been reported at about 3 and 7 days after stroke separately (Mirza et al., 2015). Microglia phenotypic shift and neuroinflammation are closely associated with the progression of stroke.

Many studies have now shown anti-neuroinflammatory effects in mouse models of stroke by modulating microglia polarization. These studies usually target TLR and Notch-related signaling pathways. In mouse/rat middle cerebral artery occlusion (MCAO) and oxygen-glucose deprivation (OGD) models, polyinosinic-polycytidylic acid activates TLR3/IRF3 signaling pathway and inhibits TLR4/NF-κB signaling pathway to reduce brain edema and improve prognosis (Wang et al., 2014). In hypoxia rat models, N-[N- (3,5-difluorophenacetyl) -1-alany1-S-phenyglycine t-butyl ester (DAPT) inhibited NF-κB/p65 expression by suppressing the Notch signaling pathway and TLR4/MyD88/TNF receptor associated factor 6 (TRAF6) pathway. Ultimately the expression levels of various inflammatory mediators such as TNF, IL-1*β* and iNOS proteins were reduced (Yao et al., 2013). In ischemia-reperfusion, NOSH-NBP, a novel mixture, can suppress the TLR4/MyD88/NF-κB pathway which leads to reduction of pro-inflammatory factors and microglia conversion to M1 phenotype. It also enhances the nuclear translocation of PPAR*γ* and promotes the M2 polarization of microglia (Ji et al., 2017). cAMP-responsive element-binding protein (CREB) competes with NF-κB signaling pathway for the same coactivator molecule. By selectively activating CREB, the expression of M2-related genes may be increased and stroke related brain injury may be improved (Xia et al., 2015).

### Microglia and Oxidative Blast

Oxidative stress is one of the important mechanisms that cause brain damage after stroke. Studies have shown that activated M1 glial cells such as microglia in the semidark zone in stroke will generate large amounts of oxidants including superoxide anion, hydrogen peroxide and peroxynitrite or nitrogen dioxide (Orellana-Urzúa et al., 2020). First, ROS lead to membrane damage and calcium overload, which in turn leads to mitochondrial dysfunction and ATP deficiency (Orellana-Urzúa et al., 2020). ATP deficiency will lead to sodium-potassium-ATPase and calcium pump dysfunction, ultimately exacerbating the disorder. In the case of stroke, oxidative stress is further exacerbated by the activation of enzyme xanthine oxidase enzyme (XO) enzymes, which further produce uric acid, superoxide radical anion and hydrogen peroxide by oxidizing hypoxanthine (Zhang et al., 2017b). In the mouse stroke model, the expression levels of antioxidants such as Superoxide dismutase (SOD) and Glutathione (GSH) are significantly reduced, followed by the exposure of proteins, lipids, and nucleic acids to ROS attack leading to neuronal cell death (Chamorro et al., 2016). Microglia located in the ischemic semidark zone of stroke are a major source of ROS (Chamorro et al., 2016). Ischemia and hypoxia lead to neuronal damage and release of DAMPs, which induce microglia to polarize to type M1. STAT family members play an important role in microglia polarization, with interferon *γ* (IFN-*γ*) activating STAT1 to promote M1 phenotype and IL-13 and IL-4 activating STAT6 to promote microglia polarization to type M2 (Allen et al., 2013). In stroke, activation of the TLR/NF-κB signaling pathway is associated with the induction of M1 microglia by IFN*γ* secreted by T helper 1 (Th1) cells (Zhao et al., 2017). NADPH oxidase 2 (NOX2) signaling activation is main pathway for ROS release from microglia. The regulatory part of NOX2 moves to the membrane and binds to the membrane-bound xanthochrome subunit upon induction by inflammatory stimuli such as IFN-*γ*, thus becoming a complex with nicotinamide adenine dinucleotide phosphate (NADPH) oxidase activity, which eventually promotes ROS production (Radak et al., 2017).

### Microglia and Neuroregeneration

M2 phenotype of microglia are thought to be beneficial for neuronal regeneration. Microglia activated to M2 phenotype by IL-4, TGF-*β* will release brain-derived neurotrophic factor (BDNF), vascular endothelial growth factor (VEGF) and insulin-like growth factor-1 (IGF-1) (Hu et al., 2015; Spejo et al., 2018). BDNF is able to promote nerve survival, recovery, and regeneration (Allen et al., 2013). In a female mouse model, BDNF was found to interact with TrkB receptors expressed by oligodendrocytes to promote myelin formation, increase oligodendrocyte differentiation and myelin thickness after demyelination injury (Fletcher et al., 2018). Currently, various methods to polarize microglia M2 to promote neuronal regeneration are vigorous. Ultrasound intervention is a new approach that has recently emerged. This approach involves engineering platelet membrane (PM) decorated resting microglia, liposome-encapsulated IL-4 (here called CPIL4) and ultrasound to specifically polarize microglia to an anti-inflammatory phenotype. Intravenous injection of this specific microglia, followed by ultrasound irradiation to remotely control the local destruction of liposomes and release of IL-4, allows local conversion of microglia to the M2 phenotype, thereby inducing regeneration of vascular and neuronal structures in mice with ischemic stroke (Liu et al., 2021). The purinergic receptor (P2X4R) has a regulatory role in the phenotypic conversion of microglia. Ivermectin (IVM) promotes myelin regeneration by enhancing the P2X4R signaling pathway to promote the phenotypic conversion of microglia to the M2 phenotype (Zabala et al., 2018). In addition, miR-124-3P-containing exosomes secreted by M2 microglia were found to promote neurite outgrowth (Huang et al., 2018). Some drugs that inhibit M1 polarization of microglia have also been found to promote neurite regeneration under pathological conditions. In a mouse model of depression, Minocycline attenuated the inhibitory effect of neurotoxic M1 microglia on neurogenesis under chronic stress (Bassett et al., 2021). M1 microglia can exert synaptic phagocytic pruning by recognizing the complement component subunit 1q (C1q) complex at neuronal synapses (Allen et al., 2013). Short-Chain Fatty Acids promote post-stroke recovery by inhibiting microglia activation and thereby mitigating synaptic elimination (Kano et al., 2019). Collectively, promoting neuronal regeneration by facilitating microglia M2 polarization has great potential for application.

### Microglia and Neuron Apoptosis

Brain tissue ischemia will result in insufficient supply of glucose and oxygen, which will disrupt the mitochondrial electron transport chain and lead to reduced ATP production (Ahsan et al., 2021). Dysregulation of ion concentrations caused by ATP deficiency will lead to a series of sequential events such as glutamate excitotoxicity. Excess glutamate will lead to calcium overload in peripheral cells, which induces neuronal cell death (Simpson and Oliver, 2020). Animal studies have shown that the expression of TNF, TNF-related inducing ligand (TRAIL), and Fas ligand (FasL) are all upregulated after ischemia, and these molecules worsen stroke prognosis to some extent (Radak et al., 2017). Microglia trigger neuronal cell death mainly through two pathways including death receptor pathway and mitochondrial pathway (Fricker et al., 2018). M1 microglia activate the death receptor pathway through the release of TNF. Through a cascade of reaction, TNF promotes activation of Bcl-2-associated X (Bid)and causes mitochondrial outer membrane permeabilization (MOMP). Ultimately, cytochrome c and caspase-9 activate downstream effectors to cleave cellular proteins and thus promote neuron apoptosis (Micheau and Tschopp, 2003; Haase et al., 2008). Many factors can activate the mitochondrial pathway, such as toxins, radiation and ROS released from M1 microglia. ROS can directly induce mitochondrial outer membrane permeabilization, which activates caspase-9 and acts downstream on caspase-3 and caspase-7, ultimately inducing apoptosis in neurons (Circu and Aw, 2010).

### Microglia Autophagy

Autophagy affects stroke progression by regulating microglia polarization. Peroxisome proliferator-activated receptor *γ* (PPAR*γ*) is a class of ligand-activated transcription factors that are important for inflammation regulation (Guo et al., 2017). It was shown that the PPAR*γ* antagonist T0070907 could inhibit the expression of M1 markers such as iNOS and promote the expression of M2 markers such as CD206. This result was shown to be associated with enhanced cellular autophagy induced by LKB1-AMPK activation (Ji et al., 2018). In addition, it has been shown that Oxiracetam (ORC) promotes microglia autophagy through the AMPK/mTOR (AMPK inhibits mTOR) pathway, thereby promoting microglia M2 polarization (Wang et al., 2021a). It has also been demonstrated that peripheral macrophage-derived exosomes (PM-Exos) can locally induce M2 microglia production by promoting autophagy (Pandey et al., 2021). Furthermore, in the mouse oxygen glucose deprivation/reperfusion (OGD/R) model, microglia autophagic flux was inhibited by suppression of NF-κB pathway. Meanwhile, rise in M1 markers and decrease in M2 markers were detected when OGD/R reached 72 h (Xia et al., 2016). The above studies suggest that autophagy plays a role in the inhibition of microglia M2 polarization and neuroinflammation, and that inhibition of microglia autophagy would exacerbate neuroinflammation. However, miR-30d-5p-capsuling exosome secreted by adipose stem cells (ADSCs) was found to significantly reduce the area of infarct injury by inhibiting autophagy and promoting M2 microglia in AIS patients and rat models and *in vitro* models of hypoxia-glucose deprivation primary microglia (Jiang et al., 2018a). Heterogeneity of these results remains to be clarified.

### Microglia Pyroptosis

In stroke, inflammasome is activated and then activates caspase-1 to shear gasdermin D, the nitrogen terminal of which form pores in the plasma membrane, finally leading to cell rupture and inflammatory responses (Tuo et al., 2021) (Fricker et al., 2018). Most recent studies have showed adverse effects of pyroptosis on the prognosis of ischemic stroke. Inhibition of platelet-activated factor receptors can inhibit the activation of down-regulated inflammasomeand thus reduce pyroptosis, while reducing ischemia-reperfusion injury (Zhao et al., 2021). In the Transient middle cerebral artery occlusion (tMCAO) mouse model, Peroxisome proliferator-activated receptor gamma coactivator 1*α* (PGC-1*α*) stimulated by medioresinol acts on the PPAR*α*/Glutamate Oxalo (GOT1) axis to significantly reduce the expression of pyroptosis-related proteins and improve ischemic brain injury (Wang et al., 2021b). Microglia pyroptosis was found in ischemia-reperfusion injury (Wang et al., 2020). Bone marrow mesenchymal stem cell-derived exosomes (BMSCs-Exos) are neuroprotective by downregulating the expression of neuronal NLR family pyrin domain containing 3 (NLRP3) inflammasome and pyroptosis-related proteins (Liu et al., 2021). In pMCAO mice, dexmedetomidine inhibited microglia pyroptosis by acting on the P2X7R/NLRP3/Caspase-1 signaling pathway and play a protective role in ischemic brain injury (Sun et al., 2021). In retinal ischemic injury, a NOD-like receptor named NLR caspase recruitment domain (CARD) containing 5 (NLRC5) was identified, which binds to the inflammasome NLRP3 and NLR-family CARD-containing protein 4 (NLRC4) and mediates microglial cell pyroptosis (Deng et al., 2021). In addition, in spinal cord injury, CD73 may inhibit the activation of the NLRP3 inflammasome complex through the adenosine-A2B adenosine receptor- phosphoinositide 3-kinase (PI3K)-AKT-Foxo1 cascade, reducing the activation of gasdermin D and thus microglia pyroptosis (Xu et al., 2021). Microglia pyroptosis and stroke nerve injury are closely related. However, reports on microglia pyroptosis in stroke models are still lacking and need to be further added. Furthermore, despite the lack of studies linking microglia polarization and pyroptosis, it is known that NLRP3 activation is associated with both microglia M1 polarization and the occurrence of pyroptosis. Therefore, the relationship between microglia pyroptosis and their pro-inflammatory polarization deserves further exploration.

### Microglia and Glutamate Excitotoxicity

Glutamate excitotoxicity is an important mechanism associated with neuronal death in stroke. Ischemia and hypoxia lead to impaired ATP synthesis, which leads to sodium-potassium pump dysfunction and eventually causes depolarization of the cell membrane and activation of some calcium channels. This process will lead to increased release and decreased reuptake of glutamate (Fricker et al., 2018). Excessive concentrations of glutamate bound to N-methyl-D-aspartate (NMDA) receptors will lead to calcium overload and thus induce peripheral neuronal death (Yang et al., 2019b). After microglia activation, large amounts of glutamate are released through two glutamate transport systems, the glutamate transporter 1 (GLT-1) transport system and the xCT transport system (Belov Kirdajova et al., 2020). It has been shown that Post-synaptic density 93 (PSD-93) binds to CX3 chemokine ligand 1 (CX3CL1) and thereby activates microglia, mediating ischemia-induced glutamate excitotoxicity in acute ischemic stroke (Zhang et al., 2021a). Glutamate excitotoxicity can also be reduced by controlling the signaling pathway of glutamate release of microglia. Inhibition of endoplasmic reticulum inositol 1,4,5-trisphosphate (InsP3) receptor activation with vitamin C improves the permeability of microglia gap junctions and inhibits glutamate release (Socodato et al., 2018). Microglia reduce glutamate excitotoxicity by modulating neuronal calcium metabolism and reducing calcium overload (Szalay et al., 2016). In addition, G protein-coupled receptor 30 (GPR30) has been demonstrated to be neuroprotective and reduce glutamate-induced excitotoxity. G1, a stimulator of GPR30, can promote microglia polarization to M2 phenotype and thus promotes the neuroprotection (Yang et al., 2021).

### Microglia and Blood Brain Barrier Protection

Disruption of the blood-brain barrier is also one of the pathological features of stroke, mainly characterized by structural disruption of tight junction protein complexes and increased permeability, which usually implicates poor prognosis (Profaci et al., 2020). P2RY12 (purinergic receptor P2Y, G-protein coupled, 12) can mediate the movement of microglia to the site of blood-brain barrier damage. These aggregated microglia can not only patch up small breaches, but they can also temporarily assume the function of the blood-brain barrier (Lou et al., 2016). When microglia are polarized to the M2 phenotype, they can better protect the blood-brain barrier by producing IL-10 and TGF-*β*1, as well as VEGF, IL-8 and pro-MMP-9 to promote vascular remodeling (Jiang et al., 2018b). The use of tetramethylpyrazine (TMP) in experimental autoimmune encephalomyelitis animal models protects the blood-spinal cord barrier by activating the signal transducer and activator of transcription 3 (STAT3)/Suppressor of Cytokine Signaling 3 (SOCS3) signaling pathway and promoting microglia polarization from M1 to M2 phenotype (Zhang et al., 2021b). M1-type microglia not only secrete pro-inflammatory cytokines, but also secrete chemokines such as chemokine (C-C motif) ligand 2 (CCL2) and Chemokine C-X-C ligand 10 (CXCL10) to recruit more immune cells and compromise the integrity of the blood-brain barrier (Jiang et al., 2018b). Minocycline may be an ideal therapeutic agent because of its good blood-brain barrier permeability, high safety profile, and targeting of M1 microglia. In a transient MCAO model, the use of minocycline promoted microglia M2 polarization significantly reduced TNF and IL-1*β* levels and increased TGF-*β*, IL-10 and chitinase-like proteins (YM1) levels, thereby reducing cerebral infarct size, promoting vascular remodeling, and improving stroke prognosis (Yang et al., 2015).

### Interplay Between Microglia and Astrocyte

**FIGURE 2:**
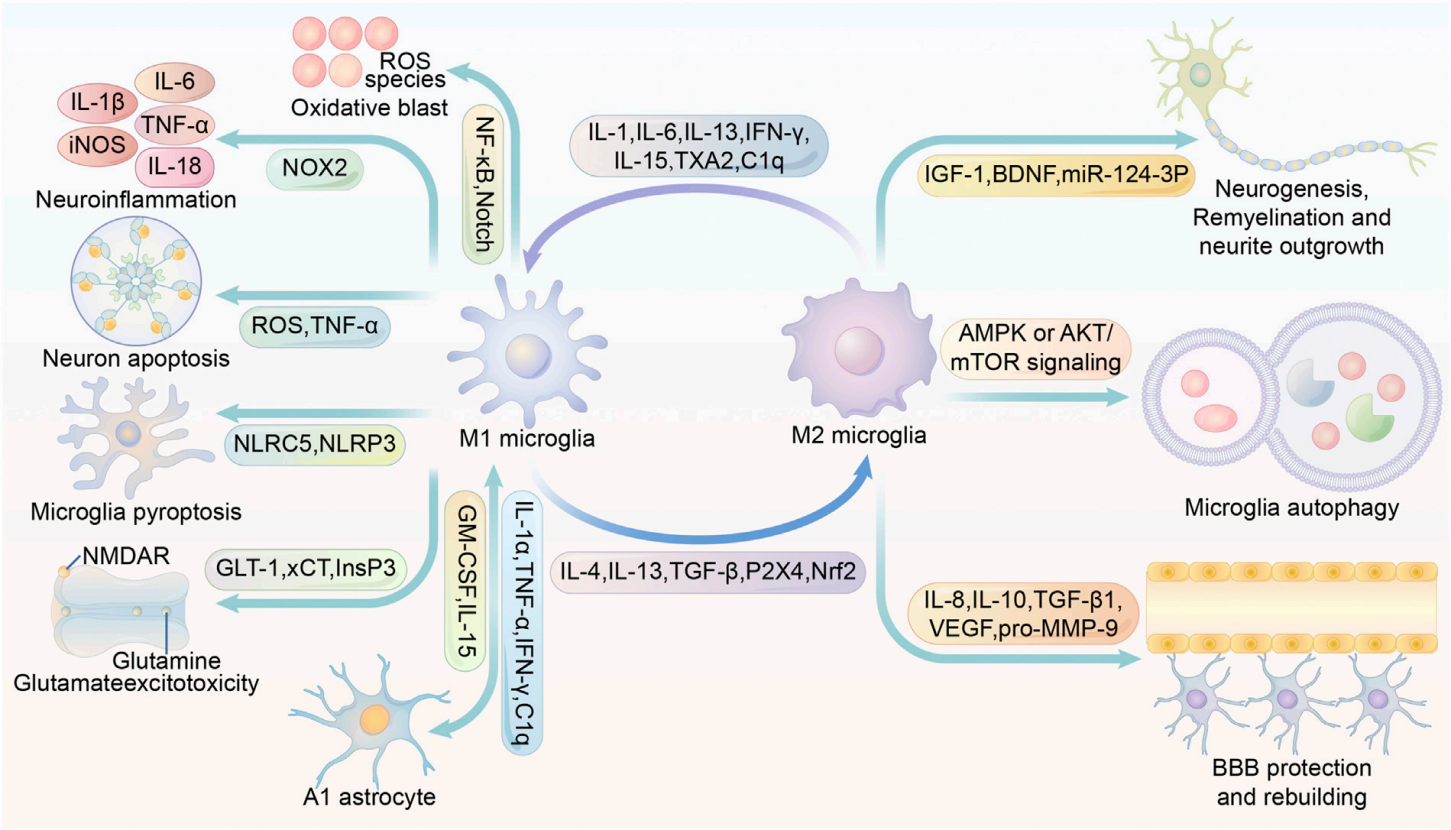
Multiple roles of M1/M2 microglia in stroke modulation. IL-1, IL-6, IL-13, IFN-*γ*, IL-15, TXA2 and C1q promote microglia M1 polarization or M2 microglia conversion to M1. IL-4, IL-13, TGF-*β*, P2X4 and Nrf2 promote microglia M2 conversion or M1 microglia conversion to M2. Activation of NF-κB and Notch in M1 microglia promotes inflammatory factor production. activation of NOX2 in M1 microglia promotes ROS production. M1 microglia are able to secrete ROS and TNF and thus induce neuronal apoptosis. activation of NLRP3 and NLRC5 in M1 microglia may contribute to microglia scorching. The inhibition of InsP3 receptors facilitates the inhibition of glutamate release. M1 microglia secrete IL-1*α*,TNF-*α*,IFN-*γ*, C1q to promote the A1 phenotype of astrocytes. A1 phenotype astrocytes secrete GM-CSF and IL-15 to promote microglia M1 polarization. glial cell M1 polarization. IL-4, IL-13, TGF-*β*, P2X4, Nrf2 induce microglia M2 polarization. M2 microglia promote neurogenesis, remyelination and neurite outgrowth by secreting IGF-1, BDNF and miR-124-3p-containing exosomes. Increased AMPK or AKT/mTOR signaling promotes microglia autophagy, and enhanced autophagy is closely associated with microglia M2 polarization. M2 microglia promote BBB protection and rebuilding by secreting IL-8, IL-10, TGF-*β*1, VEGF and pro-MMP-9. Abbreviations: iNOS, inducible nitric oxide synthase; TNF-*α*, tumour necrosis factor *α*; ROS, reactive oxygen species; NF-κB, Nuclear factor kappa B; NMDAR, N-methyl-D-aspartic acid or N-methyl-D-aspartate receptor; NOX2, NADPH oxidase 2; NLRC5, NOD-, LRR- and CARD-containing 5; NLRP3, NOD-like receptor protein 3; GLT-1, glutamate transporter-1; xCT, cystine/glutamate antiporter; InsP3, inositol 1,4,5-trisphosphate; GM-CSF, granulocyte-macrophage colony-stimulating factor; TXA2, thromboxane A2; TGF-*β*, tumor growth factor-*β*; Nrf2, Nuclear factor erythroid 2-related factor; IGF-1, insulin-like growth factor 1; BDNF, brain-derived neurotrophic factor; AMPK, AMP-activated protein kinase; mTOR, mammalian target of rapamycin; VEGF, vascular endothelial growth factor; BBB, blood-brain barrier.

IL-1*β* released from M1 microglia can promote astrocyte activation. In addition, IL-1*α*, TNF and C1q can also differentiate astrocytes into the A1 phenotype, which is detrimental to stroke recovery (Liddelow and Barres, 2017). Under physiological conditions, astrocytes can render microglia dormant, but in ischemic conditions, the ability of astrocytes to spread calcium ions over long distances may allow microglia to be activated even far from the infarct area (Nedergaard and Dirnagl, 2005). It has been shown that the use of antagonists of purinergic receptors can effectively block this transmission and slow down the expansion of the infarct volume (Xie et al., 2011). *In vitro* cultures of microglia and astrocytes were mixed. Astrocytes stimulated substantial proliferation and M2 polarization of microglia, while astrocytes appeared to differentiate to the beneficial A2 type (Kim and Son, 2021). In the stroke simulation experiment, M2 microglia and A2 astrocytes were highly susceptible to pro-inflammatory cytokines. Secretion of TNF and IFN-*γ* by microglia induced transition of A2 astrocytes to A1, and secretion of granulocyte-macrophage colony-stimulating factor (GM-CSF) by astrocytes caused M2 microglia polarization to M1 (Kim and Son, 2021). In a rat model of tMCAO, microglia-derived macrophage-like cells (MG-MΦ) upregulate astrocyte aquaporin-4 (AQP4) expression and exacerbate brain edema (Murata et al., 2020). In chronic recurrent experimental autoimmune encephalomyelitis, astrocyte-secreted TGF-*β*2 was found to exacerbate cerebral edema by possibly downregulating major histocompatibility complex class II expression and costimulatory/adhesion molecules, thereby affecting the antigen-presenting function of microglia. Besides, TGF-*β*2 secreted by prompted astrocytes appear to promote neuroinflammation (Siglienti et al., 2007). Complement C3 secreted by astrocytes can acts on neurons and microglia, resulting in impaired phagocytosis and subsequent abnormal synaptic pruning of microglia (Lian et al., 2016). In cerebral hemorrhage, IL-15 secretion by astrocytes is significantly upregulated and contributes to the microglia polarization to M1 phenotype, exacerbating brain injury (Shi et al., 2020).. (Figure 2)

## Exosome Treatment Associated With Microglia Polarization in Stroke Animal Model

Recently, a large number of studies have demonstrated that exosomes from different sources exert a significant regulatory effect on stroke-induced brain injury by targeting microglia polarization. Exosomes from endotoxemia mouse serum contain a large number of inflammation-associated miRNAs, which are phagocytosed by microglia and ultimately lead to increased neuroinflammation by promoting microglia activation and M1 polarization (Li et al., 2018). Young rat serum exosomes contain more CD46 and fewer complement components such as C1q. It was shown that serum exosomes from young rats could improve short- and long-term neurological outcomes in older rats with ischemic stroke by reducing M1 microglia and increasing M2 microglia (Zhang et al., 2021c). In acute ischemic stroke (AIS) patients and rat models, exosomes from different sources promote M2 polarization of microglia by releasing hsa-miR-124-3p and miR-30d-5p, respectively, thereby reducing brain damage caused by neuroinflammation (Jiang et al., 2018a; Qi et al., 2021). Exosomes derived from mesenchymal stem cells (MSCs) containing miR-223-3p were found to reduce infarct size and improve neurological deficits and learning memory in a mouse model of stroke by inhibiting the inflammatory response induced by M1 phenotype microglia (Zhao et al., 2020). Further studies found that exosomes from bone marrow mesenchymal stem cells (BMSCs) in Intracerebral hemorrhage (ICH) and MCAO mice reduced M1 microglia polarization and increased M2 polarization, thereby reducing neuroinflammation and neuronal apoptosis caused by ischemia or hemorrhage (Duan et al., 2020; Liu et al., 2021). In addition, human umbilical cord mesenchymal stem cells (hUMSCs) (Yang et al., 2020b; Zhang et al., 2021d), neural progenitor cells (Tian et al., 2021), lipopolysaccharide-stimulated macrophages (Zheng et al., 2019) and ADSCs (Geng et al., 2019) were found to alleviate ischemic injury caused by MCAO and promote neuroprotection and neurogenesis. These effects were shown to be closely related to exosome-induced microglia M2 polarization.

In addition to their own contained components that can exert stroke modulating effects, nanoscale exosomes can also act as carriers to transport relevant drugs or chemical molecules to modulate microglia polarization and thus influence stroke progression. Exosomes encapsulated with edaravone or melatonin can promote microglia M2 polarization and inhibit neuroinflammation (Li et al., 2020c; Liu et al., 2020). Exosomes derived from human embryonic kidney cells loaded with nerve growth factor (NGF) and its mRNA cause a decrease in the M1 phenotype and an increase in the M2 phenotype of microglia, thereby reducing ischemic damage caused by inflammation and cell death (Yang et al., 2020c). The above evidence provides a solid theoretical basis for the application of exosomes targeting microglia polarization in the treatment of stroke (Table 1).

**TABLE 1:** Therapeutic effects of exosome on stroke by targeting microglia polarization.

Exosome	Objectives	Contents	Significance	Mechanism	References
Exosomes from serum of young rats	Aged ischemic rats	More CD46, less C1q, C3a, C3b	Improved short- and long-term functional outcomes after ischemic stroke and reduced synaptic loss	Reducing Iba1^+^CD86^+^ microglia but increasing Iba1^+^CD206^+^ microglia	147
EVs from serum	AIS patients	hsa-miR-124-3p	Reduced serum pro-inflammatory cytokines and the NIHSS score	Reversing the LPS-induced inflammatory effect in BV2 microglia by inhibiting the expression of GRB2 and AKT3 gene involved in pro-inflammatory signaling pathways	148
Exosome from ADSCs	AIS rats	miR-30d-5p	Reduced cerebral injury	Suppressing autophagy and promoting M2 microglia polarization	119
Exosomes from serum	Endotoxemia mice	miR-15a, miR-15b, miR-21, miR-27b, miR-125a, miR-146a, and miR-155	Increased systemic pro-inflammatory cytokine production, and elevated CNS expression of pro-inflammatory cytokine mRNA and the inflammation-associated miR-155	Inducing the expression of Iba-1 and microglial uptake of exosomes derived from serum containing inflammation-related miRNAs	146
Exosomes from BMSCs	ICH rats	miR-146a-5p	Improved neurological function and reduced neuronal apoptosis	Inhibiting microglial M1 polarization by downregulating the expression of IRAK1 and NFAT5	75
Exosomes from hUMSCs	Ischemic mice	miR-146a-5p	Improved recovery of function, attenuated microglia-mediated inflammation	Decreasing IBA-1^+^CD16^+^ cells and increasing IBA-1^+^CD206^+^ cells by suppressing IRAK1/TRAF6 signaling pathway	151
EVs from neural progenitor cell	MCAO mice	let-7g-5p, miR-99a-5p, let-7i-5p, miR-139-5p, miR-98-5p, miR-21-5p and let-7b-5p	Suppressed inflammation response	Inhibiting the expression of Iba-1 and MAPK of an inflammation related pathway	152
Exosome from BMSCs	MCAO rats	NR	Attenuated cerebral ischemia-reperfusion injury-induced neuroinflammation and pyroptosis	Shifting M1-polarized microglia shifted toward M2-polarized microglia	104
Exosomes from macrophage	pMCAO rats	Edaravone	Enhanced neuroprotection	Promoting the polarization of microglia from M1 to M2	155
Exosomes from plasma	pMCAO rats	melatonin	Decreased infarct volume, improved recovery of function and reduced microglia pyroptosis	Inhibiting TLR4/NF-κB pathway mediated microglial inflammation and NLRP3-mediated microglia pyroptosis	156
Exosomes from hUMSCs	tMCAO rats	CCR2	Enhanced oligodendrogenesis and remyelination	Decreasing CD16 and IL-1*β* mRNA expression and increasing CD206 and Arg-1 mRNA expression	150
Exosomes from LPS stimulated macrophage	MCAO/R rats	NR	Increased neuroprotection and functional improvement	Enhancing the microglial polarization from M1 phenotype to M2 phenotype	153
Exosomes from ADSCs	MCAO/R rats	miR-126	Improved neurogenesis and functional recovery	Inhibited microglial activation and the expression of inflammatory factors	154
Exosomes from MSCs	MCAO/R rats	miR-223-3p	Decreased cerebral infarct volume, improved neurological deficits, learning and memorizing abilities	Inhibiting microglial M1 polarization mediated pro-inflammatory response	149
Exosomes from human embryonic kidney cells	Photothrombotic ischemic mice	NGF and NGF mRNA	Reduced ischemic injury via reducing inflammation and cell death	Reducing CD16^+^ M1 microglia but increasing CD206^+^ M2 microglia	157

Not reported, NR; middle cerebral artery occlusion and reperfusion, MCAO/R; bone marrow mesenchymal stem cells, BMSCs; Intracerebral hemorrhage, ICH; mesenchymal stem cells, MSCs; Cysteinyl leukotriene receptor 2, CysLT_2_R; acute ischemic stroke, AIS; adipose-derived stem cells, ADSCs; C-C chemokine receptor type 2, CCR2; transient middle cerebral artery occlusion, tMCAO; permanent middle cerebral artery occlusion, pMCAO; extracellular vesicles, EVs; lipopolysaccharide, LPS; central nervous system, CNS; complement component 1q, C1q; National Institutes of Health Stroke Scale, NIHSS; oxygen-glucose-deprivation, OGD; nerve growth factor, NGF; Intracerebral hemorrhage, ICH; Signal transducer and activator of Transcription3, STAT3; human umbilical cord mesenchymal stem cells, HUC-MSCs; ubiquitin-specific protease 14, USP14; interleukin 1 receptor associated kinase 1,IRAK1; NFAT5 nuclear factor of activated T cells 5; toll like receptor, TLR; nuclear factor-κB, NF-κB; NLR family pyrin domain containing 3, NLRP3; human umbilical cord mesenchymal stem cells, hUMSCs; TNF receptor associated factor 6, TRAF6;

Post-polarized microglia can secrete exosomes to influence stroke progression. Exosomes from microglia can promote neuronal necrosis and ICH injury by transmitting the pro-inflammatory miR-383-3p in ICH rats that sacrificed within 7 days (Wei et al., 2021). It was shown that exosomes derived from M2 microglia can reduce the volume of MCAO-induced cerebral infarcts, behavioral defects and promote neovascularization and neuronal survival by releasing miR-124, miR-137 and miR-26a within 3 days after ischemic attack (Song et al., 2019; Tian et al., 2019; Feng et al., 2021). OGD treated microglia can secrete exosomes containing miR-424-5p, which inhibit the fibroblast growth factor 2 (FGF2)/STAT3 signaling pathway and worsen endothelial cell damage (Xie et al., 2020). According to the *in vivo* study, activated microglia in the peri-infarct ischemic region were significantly increased in the first day and peaked at the 14th day after MCAO. The use of vinpocetine promotes microglia M2 polarization by enhancing microglia autophagy, ultimately attenuating neuronal damage caused by OGD treatment (Zang et al., 2020). The exosomes secreted by polarized microglia may also be an effective target for therapeutic modulation of stroke (Table 2).

**TABLE 2:** Roles of exosomes derived from polarized microglia in stroke treatment.

Exosome	Objectives	Contents	Significance	Mechanism	References
Exosomes from microglia	ICH rats	miR-383-3p	Increased necroptosis and aggravated ICH injury	Inhibiting ATF4 expression by transferring inflammation-related miR-383-3p	158
Exosomes from M2 microglia	tMCAO mice	miR-124	Attenuated ischemic brain injury and promoted neuronal survival	Inhibiting the downstream target USP14	159
Exosomes from M2 microglia	tMCAO mice	miR-137	Decreased infarct volume and behavioral deficits	Downregulating the expression of Notch-1	160
Exosomes from M2 microglia	MCAO mice	miR-26a	Promoted angiogenesis	NR	161
Exosomes from microglia	OGD conditioned microglia	miR-424-5p	Increased cerebral endothelial cell injury	Downregulating FGF2/STAT3 pathway	162
Exosomes from vinpocetine-treated microglia	OGD conditioned microglia	NR	Alleviated OGD-induced neuronal damage and influenced neuronal viability and neurite morphology	Inhibiting the OGD-Induced M1-BV2 Activation and Promoting M2 Phenotype by enhancing autophagic flux	163

Not reported, NR; oxygen-glucose-deprivation, OGD; Intracerebral hemorrhage, ICH; transient middle cerebral artery occlusion, tMCAO; C-C chemokine receptor type 2, CCR2; Fibroblast growth factor-2, FGF-2; Signal transducer and activator of transcription 3, STAT3.

## Conclusion and Outlook

Stroke remains to date a fatal and disabling serious neurological disease. Given the specificity of its lesion location and tissues and the complexity of its pathological mechanisms, the clinical outcome of stroke is still not particularly satisfactory. This is especially true for patients treated beyond the thrombolytic window. Therefore, research on new treatment modalities and therapeutic targets is particularly important.

Recent studies have shown that microglia polarization is strongly associated with stroke progression, and that M1 microglia will exacerbate stroke-induced brain injury by promoting neuroinflammation, oxidative stress, neuronal apoptosis, glutamate excitotoxicity, and astrocyte differentiation to the A1 phenotype through multiple signaling pathways. In addition, M1 microglia may further exacerbate inflammatory injury by promoting microglia pyroptosis. In contrast, M2 microglia exert a significant neuroprotective effect and promote neurological recovery. M2 microglia may promote neurogenesis, myelin regeneration, neurite growth and blood-brain barrier protection, thus exerting an important central protective function. Additionally, increased autophagic flux of microglia can promote their M2 polarization and thus reduce brain injury. Therefore, targeting microglia polarization and promoting the expression of M2 phenotype may become an important target for treatment and improving the prognosis of stroke.

Most cells are capable of secreting exosomes. Numerous studies have found that exosome pairs from different sources are able to regulate microglia polarization. This suggests that targeting the secretion of some specific exosomes may be able to regulate microglia polarization. In addition, the ability of exosomes to cross the blood-brain barrier makes them potentially an ideal vehicle for CNS drug delivery. The transport of specific drugs or molecules from exosomes to the CNS to regulate microglia polarization and improve the neurological environment has shown great potential for development. In animal models of stroke, the use of endogenous exosomes or exosomes as carriers to transport some drug molecules to promote the M2 polarization of microglia has achieved good therapeutic effects, and M2 polarized microglia can also play a corresponding neuroprotective role by secreting unique exosomes, and significantly inhibit the progression of stroke. Thus, targeting exosomes that regulate microglia and exosomes secreted by polarized microglia may be a new measure to treat stroke and improve its prognosis. However, the vast majority of exosomes have poor targeting properties. Currently, most studies have taken to implant molecules with targeting properties on the lipid membrane of exosomes, thus enhancing the targeting of exosomes. This will further increase the cost of exosome production and development, thus hindering their clinical application. Therefore, promoting the development of exosome acquisition and production technologies and increasing research on reagents that enhance exosome targeting will facilitate the clinical translation of relevant researches.
